# Transformation of Long-Lived Albino *Epipremnum aureum* ‘Golden Pothos’ and Restoring Chloroplast Development

**DOI:** 10.3389/fpls.2021.647507

**Published:** 2021-05-12

**Authors:** Chiu-Yueh Hung, Jianhui Zhang, Chayanika Bhattacharya, Hua Li, Farooqahmed S. Kittur, Carla E. Oldham, Xiangying Wei, Kent O. Burkey, Jianjun Chen, Jiahua Xie

**Affiliations:** ^1^Department of Pharmaceutical Sciences, Biomanufacturing Research Institute and Technology Enterprise, North Carolina Central University, Durham, NC, United States; ^2^Institute of Oceanography, Minjiang University, Fuzhou, China; ^3^USDA-ARS Plant Science Research Unit, Department of Crop and Soil Sciences, North Carolina State University, Raleigh, NC, United States; ^4^Environmental Horticulture Department, Mid-Florida Research and Education Center, University of Florida, Apopka, FL, United States

**Keywords:** albino, *Epipremnum aureum* ‘Golden Pothos’, Mg-protoporphyrin IX monomethyl ester cyclase, long-lived, chlorophyll biosynthesis, chloroplast development, Agrobacterium-mediated transformation

## Abstract

Chloroplasts are organelles responsible for chlorophyll biosynthesis, photosynthesis, and biosynthesis of many metabolites, which are one of key targets for crop improvement. Elucidating and engineering genes involved in chloroplast development are important approaches for studying chloroplast functions as well as developing new crops. In this study, we report a long-lived albino mutant derived from a popular ornamental plant *Epipremnum aureum* ‘Golden Pothos’ which could be used as a model for analyzing the function of genes involved in chloroplast development and generating colorful plants. Albino mutant plants were isolated from regenerated populations of variegated ‘Golden Pothos’ whose albino phenotype was previously found to be due to impaired expression of *EaZIP*, encoding Mg-protoporphyrin IX monomethyl ester cyclase. Using petioles of the mutant plants as explants with a traceable *sGFP* gene, an efficient transformation system was developed. Expressing Arabidopsis *CHL27* (a homolog of *EaZIP*) but not *EaZIP* in albino plants restored green color and chloroplast development. Interestingly, in addition to the occurrence of plants with solid green color, plants with variegated leaves and pale-yellow leaves were also obtained in the regenerated populations. Nevertheless, our study shows that these long-lived albino plants along with the established efficient transformation system could be used for creating colorful ornamental plants. This system could also potentially be used for investigating physiological processes associated with chlorophyll levels and chloroplast development as well as certain biological activities, which are difficult to achieve using green plants.

## Introduction

In plants, chloroplasts are important organelles, responsible for producing pigments, harvesting solar energy and generating various metabolites (Block et al., 2007; Terry and Smith, 2013; Pogson et al., 2015). Chloroplast biogenesis and functions require 2,500–3,500 proteins (Peltier et al., 2002; Block et al., 2007; Woodson and Chory, 2008), but its genome only encodes ∼100 proteins (Martin et al., 2002). The remaining over 95% of proteins are encoded by nuclear genes, which are synthesized in the cytosol and then imported into proplastids for chloroplast biogenesis, development, and function (Abdallah et al., 2000; Kleine et al., 2009; Pogson et al., 2015). Thus, understanding the roles of individual genes encoded by two separate genomes, their coordinated expressions, and the translocation of nuclear-encoded proteins during chloroplast development is fundamentally important for genetic engineering of plants in order to improve photosynthetic efficiency, plant growth, and seed and natural product production (Woodson and Chory, 2008; Bölter, 2018; Zoschke and Bock, 2018; Boehm and Bock, 2019).

So far, determining the roles of most nuclear-encoded plastid genes and elucidating mutual regulation of genes from two genomes during the chloroplast biogenesis and development remain big challenges because of the complexity and the lack of an effective experimental platform. Various experimental systems, such as dark-grown etiolated seedlings, various types of color-defective mutants including albino, variegated, and chemical/physical induced mutants, have been used for aforementioned studies (McCormac and Terry, 2004; Yu et al., 2007; Pogson et al., 2015; Zoschke et al., 2017). Among them, etiolated seedlings have been widely used to study the chloroplast biogenesis by monitoring the greening process in cotyledon cells upon exposure to light (Sinclair et al., 2017; Gommers and Monte, 2018; Armarego-Marriott et al., 2020). However, the transition from proplastids to chloroplasts is very quick and complex (Block et al., 2007; Woodson and Chory, 2008; Pogson et al., 2015; Bölter, 2018; Zoschke and Bock, 2018), and the expression of genes from both plastid and nuclear genomes is sharply induced (Jiao et al., 2007; Chen et al., 2010; Jing and Lin, 2020), which poses a problem to study spatially and temporally coordinated expressions of these genes during the chloroplast biogenesis.

Albino plants are also commonly used for studying chloroplast development and function. Previous studies show that albino plants derived from mutation together with their green counterparts are ideal genetic materials for identifying mutated genes involved in the chloroplast biogenesis and development. This has been demonstrated in a variety of albino mutants, such as barley *albostrians* (Hess et al., 1994; Li et al., 2019), maize *iojap* (Han et al., 1992), and *white seedling 3* (Hunter et al., 2018), Arabidopsis *seedling plastid development1* (Ruppel et al., 2011), as well as T-DNA insertion mutants Arabidopsis *pap3, pap6*, and *pap7* (Steiner et al., 2011), rice *albino Leaf1* (Zhang et al., 2016) and *albino Leaf2* (Liu et al., 2016), and tomato *wls-2297* (García-Alcázar et al., 2017). Albino mutants are also considered as ideal materials for studying mechanisms underlying chloroplast biogenesis and development (Dunford and Walden, 1991; Pfalz and Pfannschmidt, 2012; Hayashi-Tsugane et al., 2014; Yang et al., 2019). Some of them have been used to shed the light on pathways involved in chloroplast biogenesis, such as the retrograde signaling (Bradbeer et al., 1979; Börner, 2017), plastid protein import machinery (Shipman-Roston et al., 2010; Li et al., 2019), and light signal regulated genes in albino plants (Grübler et al., 2017). Chemically induced and tissue culture-derived albino plants have been used to understand the development of stomatal complex (Hernández-Castellano et al., 2020), stomatal opening and functioning (Roelfsema et al., 2006), the role of blue or red light in regulating flowering (Jabben and Deitzer, 1979; Bavrina et al., 2002), and the effects of carotenoid-derived molecules on root development patterning (Van Norman et al., 2014). Although albino plants occur in nature and can also be obtained in laboratory through somaclonal variation by cell and tissue culture, induction by physical and chemical mutagenesis, and genetic engineering, a distinct characteristic of the albino plants is that the majority of them are usually short-lived even though they are maintained on culture media with high sucrose (Dunford and Walden, 1991; Zubko and Day, 1998; Ruppel et al., 2011; Steiner et al., 2011; García-Alcázar et al., 2017). The short-lived nature of these plants is a shortcoming for their applications as an experimental platform to study the coordinated expressions of nuclear and plastid genes.

**FIGURE 1:**
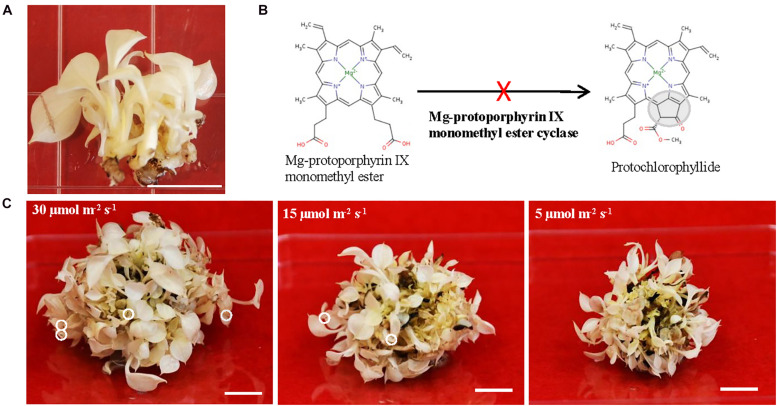
The growth of albino plants and the involvement of *EaZIP*. **(A)** Albino plantlets. **(B)** Impaired *EaZIP* in chlorophyll biosynthesis pathway. **(C)** Albino plantlets were grown under ∼30, 15, and 5 μmol m^− 2^ s^− 1^ light intensity at 23°C for 4 weeks. The white circle indicates damaged leaf-tip. Bar = 1 cm.

Long-lived albino plants having capacity to be switched from aberrant plastids to functional chloroplasts are desirable. Because they could be used as an experimental platform for investigating genes related to the chloroplast development and monitoring the translocation of nuclear-encoded plastid proteins during the greening process. Moreover, long-lived albino plants could be useful to solve certain biological questions where chlorophyll effects should be avoided. Recently, plants are emerging as alternative expression system to replace mammalian one for producing a diverse range of biopharmaceuticals (Xu et al., 2012; Moon et al., 2020). The long-lived albino plants can also be an ideal expression system to express high-value compounds and therapeutic proteins to avoid chlorophyll interference during the purification process (Wilken and Nikolov, 2012; Mellor et al., 2018). However, there are no reports on long-lived albino plants with exception of three recent studies from perennial Agave (*Agave angustifolia* Haw.) in which created albino plants are surely long-lived even though their life span was not indicated (Duarte-Aké et al., 2016; Us-Camas et al., 2017; Hernández-Castellano et al., 2020). One possibility for the lack of long-lived albino plants could be due to the life span of their parental species since most of albino mutants are derived from either annual or biennial plant species. Thus, we postulated that using perennial, vegetatively growing, non-flowering and long-lived pothos plants (*Epipremnum aureum*) (Hung and Xie, 2009; Hung et al., 2016) to develop albino plants could be a better choice. To this end, we regenerated albino plants from a variegated variety ‘Golden Pothos’ by tissue culture techniques (Hung and Xie, 2009). Regenerated albino plants (Figure 1A) have been propagated and maintained for more than 11 years in our laboratory despite being rootless. Our previous studies indicated that they have impaired expression of *EaZIP* encoding Mg-protoporphyrin IX monomethyl ester (MPE) cyclase, a key enzyme in the chlorophyll biosynthesis pathway (Figure 1B; Hung et al., 2010). Impaired expression of MPE cyclase is thought to cause defective leaf color and result in colorless plastids in various plant species (Tottey et al., 2003; Liu et al., 2004; Rzeznicka et al., 2005; Hung et al., 2010).

In view of the importance of MPE cyclase, we hypothesized that compensating *EaZIP* expression in regenerated albino plants would potentially synthesize sufficient chlorophylls for plastid development and restore the normal green phenotype. In the present study, we first expressed traceable *sGFP* gene (Chiu et al., 1996) in albino plants for developing a transformation method. We then overexpressed *EaZIP* driven by a constitutive promoter CaMV35S (*35S:EaZIP*) in albino plants to restore the normal green phenotype. However, it did not generate any green transgenic plants. When Arabidopsis *CHL27*, a homolog of *EaZIP*, driven by the CaMV35S promoter (*35S:CHL27*) was expressed, both the defective color and dysfunctional chloroplast development were restored in albino plants. Intriguingly, expressing *CHL27* in albino plants produced various leaf colors of regenerated transgenic plants. For ornamental plants, color is one of the most important traits to add value to a new cultivar (Chen et al., 2005). Generally, colorful ornamental plants are produced from green plants via natural or artificial mutation, somaclonal variation, or genetic engineering (Marcotrigiano, 1997; Van Harten, 1998; Chen and Henny, 2006; Zhao et al., 2012). The present study presents a new strategy for producing colorful plants by genetically engineering of long-lived albino plants. Potential mechanisms of developing different color plants and application of long-lived albino plants were discussed.

## Materials and Methods

### Plant Maintenance

Albino plantlets were derived from variegated leaves of *E. aureum* ‘Golden Pothos’ by tissue culture techniques (Hung and Xie, 2009). They were propagated in a maintenance medium containing MS salts with vitamins (Research Products International, Mount Prospect, IL, United States), 10 mg/L ascorbic acid, 25 g/L sucrose, and 5 g/L agar with pH adjusted to 5.6 and grown at 23°C under a constant light intensity of 5 μmol m^–2^ s^–1^. The plantlets were maintained by transferring onto fresh medium once a month. To observe the effects of light intensity on their growth, equal-sized clusters of albino plantlets were cultured on the maintenance medium at 23°C under full spectrum light with the intensities of 30, 15, and 5 μmol m^–2^ s^–1^, respectively for a month before they were photographed. Regenerated green transgenic plants were transplanted to soil after rooting.

### Creation of Genetic Cassettes

The *sGFP* genetic cassette, CEJ899, was kindly provided by Professor Deyu Xie of North Carolina State University. The *EaZIP* genetic cassette, CEJ1260, was created by replacing *GUS* in commercial vector pBI121 (Clontech, Mountain View, CA, United States) with *EaZIP* coding region (Accession #: FJ666046) (Hung et al., 2010). The *CHL27* genetic cassette, CEJ1264, was created by replacing *GUS* in pBI121 with *Arabidopsis thaliana CHL27* coding region (Accession #: NM_115553) (Salanoubat et al., 2000). The sequence alignment of cDNAs of *EaZIP* and Arabidopsis *CHL27* is presented in Supplementary Figure 1. After cloning, both inserted genes were sequenced for validation before used for transformation. Each plasmid DNA was introduced into *Agrobacterium tumefaciens* strain LBA4404 using the freeze–thaw method (Holsters et al., 1978).

### Plant Transformation

For *Agrobacterium*-mediated transformation, our previous transformation system developed for green *E. aureum* ‘Jade’ (Zhao et al., 2013b) was modified. Briefly, petiole segments were excised from albino plantlets and infected with *Agrobacterium* solution (OD_600_ of 0.8) containing 100 μM acetosyringone for 10 min. Then infected petiole segments were co-cultured on induction medium containing MS salts with vitamins (Research Products International), 2 mg/L *N*-(2-chloro-4-pyridl)-*N’*-phenylurea (CPPU), 0.2 mg/L α-naphthalene acetic acid (NAA), 10 mg/L ascorbic acid, 25 g/L sucrose, and 5 g/L agar (pH 5.6) in the dark. After 7-day co-culture, they were transferred onto the selection medium which is the induction medium with additional 75 mg/L kanamycin and 100 mg/L Timentin. Only one callus from each explant was isolated for the regeneration. Induced calli were transferred onto regeneration medium which is the selection medium without hormones.

### Genomic DNA PCR, RT-PCR, and qRT-PCR

Shoots maintained on regeneration medium were harvested and stored at −80°C for genomic DNA and total RNA isolation. The DNeasy Plant Mini Kit (Qiagen, Germantown, MD, United States) was used for isolating genomic DNA. The RNeasy Plant Mini Kit (Qiagen) was used for isolating total RNA. Further treatment of DNase I and first strand cDNA synthesis were performed using SuperScript^TM^ IV VILO^TM^ master mix with ezDNase^TM^ enzyme (Invitrogen, Carlsbad, CA, United States). For PCR and RT-PCR, the reactions were carried out with *Taq* DNA polymerase (Sigma Aldrich, St. Louis, MO, United States) and specific primers: NPTIIF 5′-AAGATGGATTGCACGCAGGTTC-3′ and NPTIIR 5′-ACGGGTAGCCAACGCTATGTC-3′ for the *nptII*; GFPF 5′-GAGCTGGACGGCGACGTAAA-3′ and GFPR 5′-GTGTCGCCCTCGAACTTCAC-3′ for the *GFP*; EaZIPF 5′-ACGAAGGCTAGGCAGTACAC-3′ and NosR1 5′-AAATGTATAATTGCGGGACTCT-3′ for the *EaZIP*; and CHL27F 5′-ACAACCAGACACATTTCGTGA-3′ and CHL27R 5′-ACGTCGACGAGCTCCTAATAGA-3′ for the *CHL27*. For qRT-PCR, the procedure was the same as described previously (Hung et al., 2016), while the specific primers are EaZIP-qF 5′-AGACTGAAGACATTCCCCTGGTAA-3′ and EaZIP-qR 5′-CTCAGAGACTAGTGCTGCGATGA-3′ for the *EaZIP*; and CHL27-qF 5′-GTGGTTCGGTTTGTCTCGAT-3′ and CHL27-qR 5′-ACGTCGACGAGCTCCTAATAGA-3′ for the *CHL27*. The QuantumRNA^TM^ 18S Internal Standards (Ambion, Austin, TX, United States) was used as an endogenous control.

### GFP Imaging and Transmission Electron Microscopy (TEM) Analysis

The GFP fluorescence detected in transgenic plants were captured under fluorescence microscope (Keyence, Osaka, Japan). To observe the chloroplast ultra-structures in leaf tissues, TEM analysis was performed at the Center for Electron Microscopy of North Carolina State University. Fully expanded young leaves from baby jar grown plants were cut into 1 mm^3^ blocks and then fixed in 3% glutaraldehyde in 0.05 M KPO_4_ buffer, pH 7 at 4°C. All samples were rinsed in three 30-min changes of cold 0.05 M KPO_4_ buffer, pH 7, then post-fixed in 2% OsO_4_ in the same buffer at 4°C in the dark. After dehydrated with graded series of ethanol (30, 50, 70, 95, and 100%), samples were infiltrated with Spurr’s resin (Ladd Research Industries, Williston, VT, United States), flat embedded and cured at 70°C overnight. Samples were sectioned with a Leica UC6rt ultramicrotome (Leica Microsystems, Wetzlar, Germany) and placed onto 200-mesh grids. The grids were then stained with 4% aqueous uranyl acetate in the dark at 25°C followed by three times of warm distilled water (40°C) washes and 1 min in Reynold’s lead citrate followed by three more warm distilled water washes. All sections were observed under a JEOL JEM 1200EX transmission electron microscope (JEOL, Peabody, MA, United States). Images were captured using a Gatan Erlangshen Model 785 ES1000W camera and Digital Micrograph software (Gatan, Pleasanton, CA, United States).

### Immunoblotting Analysis

For detecting the presence of GFP protein in transgenic lines, the total proteins were first extracted from mature leaf tissues using the Plant Total Protein Extraction kit (Sigma-Aldrich). The protein extract was then subjected to SDS-PAGE. They were heated with NuPAGE^TM^ LDS sample buffer containing 10% reducing agent (500 mM DTT) at 70°C for 10 min, then analyzed under a NuPAGE^TM^ 4–12% Bis-Tris gel with a MES SDS running buffer containing antioxidant as instructed by the manufacturer. For immunoblotting, proteins were transferred onto a PVDF membrane (Bio-Rad, Hercules, CA, United States). The membrane was then blocked at 25°C for 1 h with 15% (w/v) dry milk dissolved in PBST. It was then incubated at 25°C for 1 h with 1:200 diluted primary antibody anti-GFP (B-2) (sc-9996; Santa Cruz Biotechnology, Dallas, TX, United States) followed by incubation in the secondary antibody Goat anti-Rabbit IgG horseradish peroxidase conjugate (Bio-Rad). The luminescent signals were generated after incubation with SuperSignal^®^ West Pico Chemiluminescent substrate (Pierce biotechnology, Rockford, IL, United States) and captured by Kodak Biomax X-ray film (PerkinElmer, Waltham, MA, United States). For staining the PVDF membrane, 0.2% (w/v) Amido Black 10B (MP Biomedicals, Santa Ana, CA, United States) in 10% (v/v) acidic acid was used.

### Measurement of Chlorophylls

For extracting chlorophylls, the mature leaf tissues were first ground in liquid nitrogen then resuspended in 80% acetone as described in Hung and Xie (2009). After centrifugation, the clear supernatant was collected and diluted 10x in acetone before reading their absorption wavelengths using SpectraMax Plus 384 (Molecular Devices, San Jose, CA, United States). The chlorophyll content was calculated based on the formula described by Hanfrey et al. (1996). All results were presented as the average of three biological replicates ± SD. Statistical significance was analyzed by comparing all pairs using Tukey-kramer HSD (*p* < 0.05).

## Results

### Albino Plants Are Long-Lived and Grow Well Under Low Light

To explore the potential applications of regenerated albino plants, we optimized their growth conditions in order to have healthy plants for downstream studies. They were propagated and maintained on Murashige and Skoog (MS) medium (Murashige and Skoog, 1962) with 25 g/L sucrose as a carbon source and addition of 10 mg/L ascorbic acid. Ascorbic acid was used as an antioxidant agent, which is due to the notion that albino plants lacking functional chloroplasts are unable to carry out photosynthesis and more sensitive to the photo-oxidation (Spoehr, 1942; Hess et al., 1994; García-Alcázar et al., 2017). Initially, regenerated plants from color defective leaf sectors were pale yellow and had rooting capacity (Hung and Xie, 2009). However, they gradually lost pale yellow color and rooting capacity, and became albino plants without roots (Figure 1A). We found that they grew slowly but well under low light (5 μmol m^–2^ s^–1^) on MS medium with addition of 10 mg/L ascorbic acid. Although growths were much faster under high light conditions (15 and 30 μmol m^–2^ s^–1^), there were signs of cell damage on the tip of leaves (Figure 1C). Because of the high light associated cell damage, low light conditions were chosen for growth and maintenance. Since then they have grown and been successfully propagated for more than 11 years and could be considered as long-lived albino plants in comparison with other known albino plants (Hess et al., 1994; Ruppel et al., 2011; Liu et al., 2016; Zhang et al., 2016; García-Alcázar et al., 2017).

### Albino Plants Can Be Easily Transformed With a High Transformation Efficiency

**FIGURE 2:**
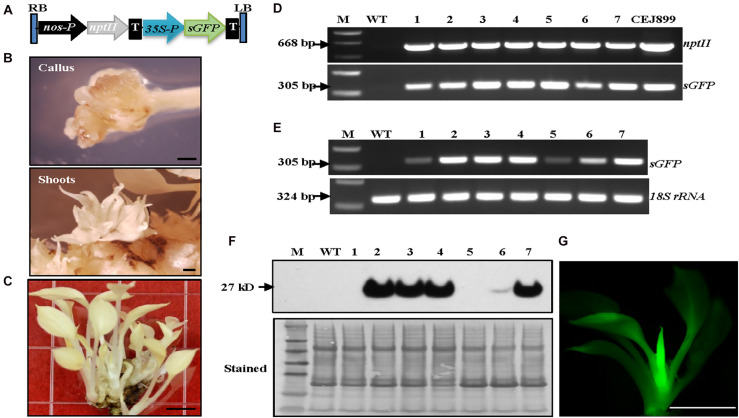
Establishment of *Agrobacterium*-mediated transformation system in albino plants expressing *sGFP.* **(A)** Genetic cassette CEJ899 with *sGFP* driven by CaMV35S promoter (35S-P) and *nptII* driven by *nos* promoter (nos-P). T, nos terminator; RB, right border; LB, left border. **(B)** Induced calli and shoots. Bar = 1 mm. **(C)** Regenerated transgenic plantlets. Bar = 5 mm. **(D)** Genomic DNA PCR and **(E)** RT-PCR of seven individual transgenic lines (1–7) amplified by specific primer pairs for *nptII* and *sGFP*, respectively, using *18S rRNA* as an internal control. M, molecular marker; WT, wild-type albino plant. **(F)** Immunoblot against anti-GFP. Stained blot shows equal protein loading. **(G)** GFP fluorescence of a transgenic plantlet. Bar = 5 mm.

After establishing the optimal growth conditions for the albino plants, a transformation system to produce stable albino transgenic plants was developed. We adopted our previous *Agrobacterium*-mediated transformation system developed for green pothos plants (Zhao et al., 2013b) with three major changes. Briefly, *nptII* was used to replace *hpt* as a selection gene (Figure 2A), 75 mg/L kanamycin was used for screening transgenic plants, and ascorbic acid was supplemented into induction, selection and regeneration media to prevent oxidative damage.

Transformation results showed that visible kanamycin resistant calli first appeared after 4 weeks on selection medium (Figure 2B). The average callus induction rate observed after 8 weeks of subculture was ∼52% (Table 1), which was higher than green pothos plants (Zhao et al., 2013b). After growing for an additional 4–6 weeks, calli were transferred onto a regeneration medium. After about 2 weeks, newly regenerated white shoots appeared (Figure 2B). To confirm the presence of transgenes *sGFP* and *nptII* in these regenerated white shoots, seven independent kanamycin-resistant lines (Figure 2C) were examined by PCR and the results showed that both genes were detected in all lines (Figure 2D). To confirm the expression of *sGFP*, RT-PCR and immunoblotting analyses were performed. The expression of *sGFP* was confirmed in all seven lines by RT-PCR (Figure 2E). Immunoblotting results based on equal loading of total proteins further confirmed the presence of GFP protein in five out of seven lines which had high transcript levels (Figure 2F). The GFP fluorescence was also observed in regenerated plants (Figure 2G). These results indicate that albino plants can be stably transformed with a high transformation efficiency.

**TABLE 1:** The induction rates of kanamycin-resistant calli and subsequently regenerated shoots.

Transgene	Batch	No. of explants	No. of explants with calli	Callus induction rate (%)	Average ± SD (%)	No. of tested calli*	No. of tested calli with green shoots	No. of tested calli with white shoots	Shoot induction rate (%)
*sGFP*	I	62	33	53.2					
	II	35	10	28.6	51.7 ± 22.4	17 (26%)	0	17	100
	III	30	22	73.3					
*EaZIP*	I	32	21	65.6					
	II	36	29	80.6	69.2 ± 10.1	31 (45%)	0	31	100
	III	31	19	61.3					
*CHL27*	I	40	35	87.5					
	II	35	30	85.7	88.1 ± 2.8	22 (23%)	17	5	100
	III	34	31	91.2					

*Calli obtained from batch I, II and III were randomly selected for the shoot induction experiment.

### Overexpressing *EaZIP* Did Not Change the Albino Phenotype

**FIGURE 3:**
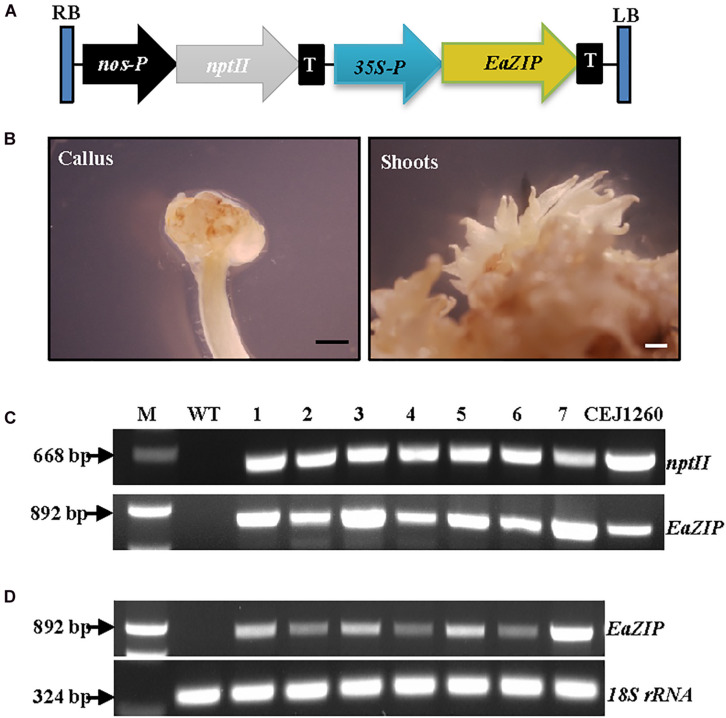
Overexpression of *EaZIP* in albino plants. **(A)** Genetic cassette CEJ1260 with *EaZIP* driven by CaMV35S promoter (35S-P). The remaining annotations are the same as in Figure 2A. **(B)** Induced calli and shoots. Bar = 1 mm. **(C)** Genomic DNA PCR and **(D)** RT-PCR of seven individual transgenic lines (1–7) amplified by specific primer pairs for *nptII* and *EaZIP*, respectively, using *18S rRNA* as an internal control. M, molecular marker; WT, wild-type albino plant.

Our previous study suggested that no expression of *EaZIP* could be responsible for the loss of green color in albino plants (Hung et al., 2010). We found that the albino plants accumulated MPE, the substrate of the MPE cyclase, when they were fed with 5-aminolevulinic acid, the first committed intermediate of the porphyrin synthesis pathway. These results indicate that the albino plants lack functional MPE cyclase. Our previous cloning effort obtained an *EaZIP* from green pothos (Hung et al., 2010), which lacks a sequence region encoding a *N*-terminal chloroplast transit peptide (cTP). When overexpressing the cloned *EaZIP* in wild type tobacco plants which carry the *NtZIP* also lacking 5′-end sequence coding for a cTP (Liu et al., 2004), surprisingly we found transgenic plants showing variegated phenotype (Guan et al., 2017). However, whether the cloned *EaZIP* is a functional version or not has not been tested. To determine if overexpression of the *EaZIP* could restore green color, we first overexpressed *EaZIP* in albino plants (Figure 3A).

Following *EaZIP* overexpression, no green shoots were observed among all regenerated plantlets. These regenerants were derived from 31 independent kanamycin-resistant calli grown with or without light exposure (Table 1), even though the growth of kanamycin-resistant calli and regenerated shoots was robust (Figure 3B) with a high transformation rate of 69% (Table 1). To confirm the presence and expression of transgenes in these regenerated plantlets, PCR and RT-PCR were performed. The presence and expression of *EaZIP* were confirmed in all selected seven lines (Figures 3C,D). The finding that overexpressing *EaZIP* was unable to restore the chlorophyll production complements with our previous study that overexpressing the *EaZIP* caused normal green tobacco plants to become variegated (Guan et al., 2017). The results from these two studies implied that the cloned *EaZIP* might be defective. One possible reason might be the lack of cTP. For further testing, using a functional Arabidopsis *CHL27* with a cTP (Tottey et al., 2003) could be an alternative.

### Introducing Arabidopsis *CHL27* in Albino Plants Restored Chlorophyll Biosynthesis

**FIGURE 4:**
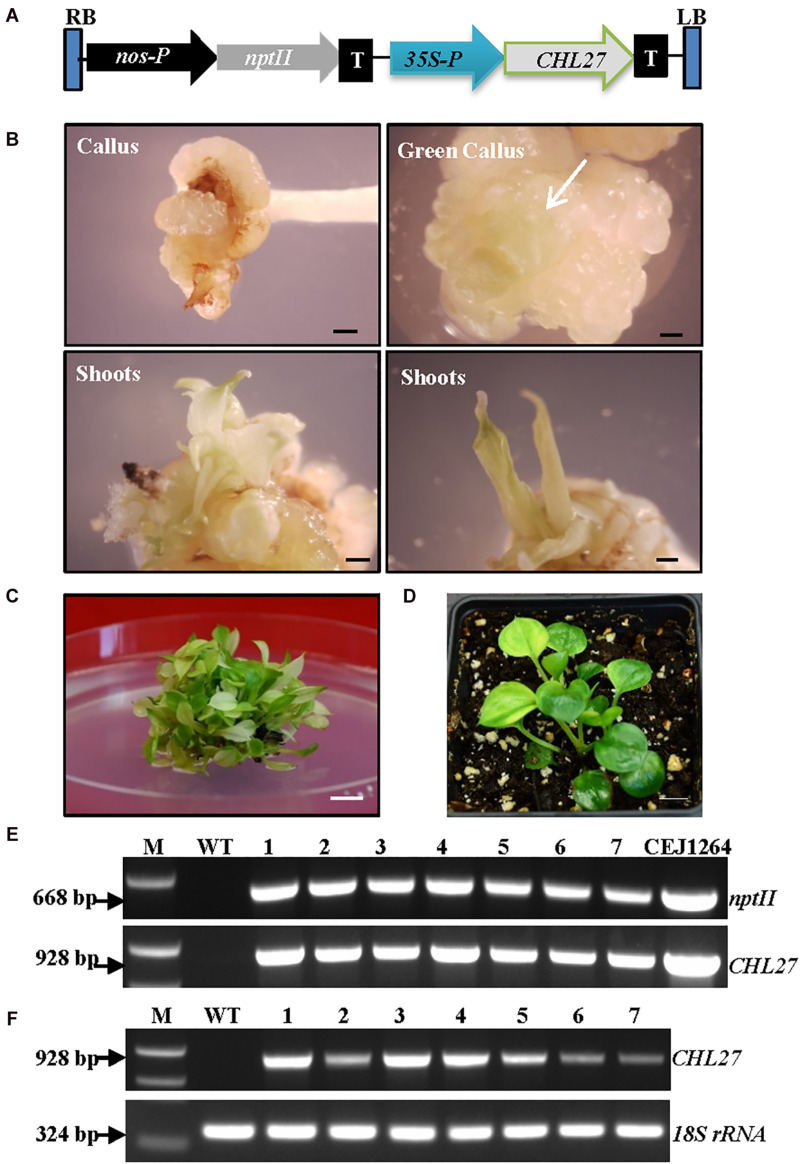
Expression of *CHL27* in albino plants. **(A)** Genetic cassette CEJ1264 with *CHL27* driven by CaMV35S promoter (35S-P). The remaining annotations are the same as in Figure 2A. **(B)** Induced calli from petiole explant, and propagated green calli (white arrow) and shoots. Bar = 1 mm. **(C)** Mixed green and pale yellow shoots. Bar = 1 cm. **(D)** Regenerated green *CHL27* transgenic plant. Bar = 1 cm. **(E)** Genomic DNA PCR and **(F)** RT-PCR of seven individual transgenic lines (1–7) amplified by specific primer pairs for *nptII* and *CHL27*, respectively, using *18S rRNA* as an internal control. M, molecular marker; WT, wild-type albino plant.

The function of Arabidopsis *CHL27* has been fully characterized (Tottey et al., 2003). We reasoned that expressing *CHL27* in albino plants might restore chlorophyll biosynthesis. Positive results would demonstrate that the impaired expression of *EaZIP* could be responsible for albino phenotype. To test this hypothesis, we created a genetic cassette by replacing *EaZIP* with *CHL27* (Figure 4A). The transformation rate was high at ∼88% (Table 1). When 22 kanamycin-resistant calli with 0.5–1 cm diameters were exposed to light for approximately 2 weeks, cells with green color started to appear (Figure 4B), and subsequently green shoots emerged (Figure 4B) in 17 individual calli (Table 1). Rooting capacity was also recovered in *CHL27* transgenic plants, which allowed them to grow well in soil (Figure 4D). To confirm these green shoots (Figure 4C) expressing *CHL27*, PCR, and RT-PCR were performed in seven lines. The PCR and RT-PCR results showed that all green shoots carried (Figure 4E) and expressed (Figure 4F) *CHL27*. These results confirm that the cTP is required and a functional form of *EaZIP* with cTP may be present in green pothos cells.

### The Leaf Color and Chlorophyll Levels of Created Transgenic Plants Correlate Well With the Expression of *CHL27*

**FIGURE 5:**
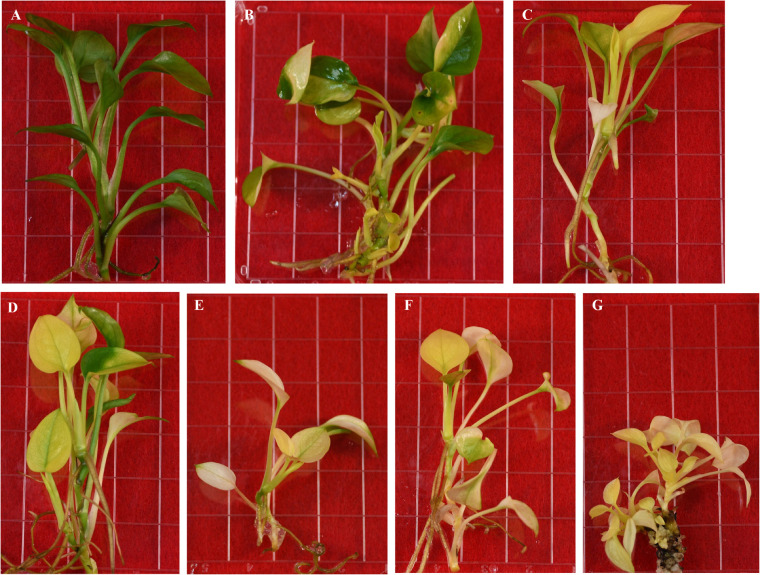
Regenerated transgenic plants from the same transformed callus expressing Arabidopsis *CHL27*. **(A)** Pure green. **(B–D)** Variegated. **(E–G)** Pale yellow.

**FIGURE 6:**
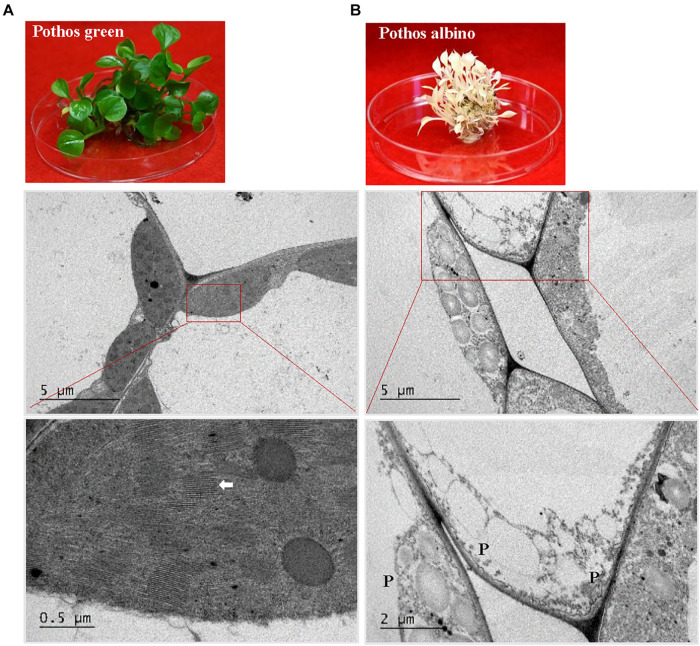
TEM analysis of chloroplast development in ‘Pothos’ wild type leaf cells. **(A)** ‘Pothos’ green and **(B)** albino plants from tissue culture (above) and TEM of their leaf cells (below). Red lines indicate the enlarged area. White arrow: detailed thylakoids; P: plastid.

**FIGURE 7:**
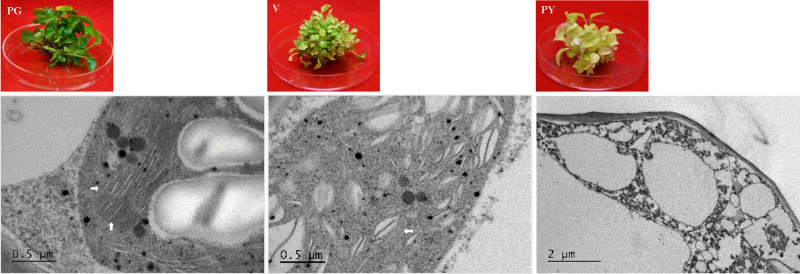
TEM analysis of chloroplast development in ‘Pothos’ transgenic leaf cells. *CHL27* transgenic plants from single transformation event with pure green (PG), variegated (V) and pale yellow (PY) leaf colors (left), and TEM (right) of their thylakoids (marked by white arrows).

We observed that the majority of regenerated shoots derived from the same transgenic callus had three types of leaves: pure green (PG), variegated (V), and pale yellow (PY) (Figure 4C). Different leaf colors appeared in regenerated plants were relatively stable when they grew under the same growth conditions (Figure 5). This phenomenon, however, did not appear in tissue culture regenerated pothos green and albino plants (Figure 6) where calli derived from green or albino explants generated uniform green or white color shoots. To determine whether the leaf color correlates with the expression of *CHL27*, three types (PG, V, and PY) of transgenic plants from the same transgenic line (Figure 7) were used to measure the contents of chlorophylls and quantify the expression levels of *CHL27*. Their ultra-structures of leaf cells were also examined since chlorophylls are required for chloroplast biogenesis, such as for assembly and maintenance of photosynthetic apparatus and stacking of thylakoid membranes (Eckhardt et al., 2004; Block et al., 2007).

The results showed that the chlorophyll *a* and *b* levels in PG transgenic plants were 3.4 and 1.25 mg/g FW, respectively (Table 2). They were elevated from undetectable in initial wild-type albino plants to 64% of levels found in pothos green plants regenerated from tissue culture. Although the total contents of chlorophylls were less, the chlorophyll *a*/*b* ratio was the same as in pothos green plants (Table 2). The chlorophyll *a* and *b* levels of V (1.72 and 0.86 mg/g FW) and PY (0.28 and 0.03 mg/g FW) transgenic plants were lower than those of pothos green and PG transgenic plants, but higher than those of wild-type albino plants. In addition, the chlorophyll *a/b* ratios of PY transgenic plants were in average of 8.14 which was abnormal compared to green plants ranging around 2.7 (Table 2). The stable chlorophyll *a*/*b* ratio is important and thought to be very critical to acclimate to changes in light conditions (Rudiger, 2002). When the expression levels of *CHL27* were quantified by qRT-PCR in three types of transgenic plants as well as wild-type green and albino plants, the levels of PG transgenic plants were 3.7- and 6.2-fold higher than those of V and PY plants, respectively (Table 3). As for pothos endogenous *EaZIP*, qRT-PCR result showed that comparing to pothos green plants, none of the wild type albino and *CHL27* PG, V and PY transgenic plants had detectable *EaZIP* (Table 3). These results further indicate that transgenic plants with different colors correlate well the restored levels of chlorophylls, which were indeed contributed from the level of *CHL27* expression.

**TABLE 2:** Chlorophyll *a* and *b* contents*.

FW (mg/g)	Pothos green	Pothos albino	PG	V	PY
Chl *a*	5.32 ± 0.80^a^	0.01 ± 0.01^d^	3.40 ± 0.64^b^	1.72 ± 0.08^c^	0.28 ± 0.05^d^
Chl *b*	1.94 ± 0.06^a^	0.01 ± 0.01^d^	1.25 ± 0.22^b^	0.86 ± 0.05^c^	0.03 ± 0.02^d^
Chl *a* + *b*	7.26 ± 0.83^a^	0.02 ± 0.02^d^	4.65 ± 0.85^b^	2.59 ± 0.11^c^	0.31 ± 0.07^d^
Chl *a*/*b*	2.74	0.59	2.71	1.99	8.14

*Data represent the average of biological replicates ± SD (n = 3). Different letters represent significant differences at p < 0.05 level.

**TABLE 3:** Quantitative RT-PCR of *CHL27* and EaZIP*.

Samples	*CHL27* Δ Ct (n = 3)	Fold changes	*EaZIP* ΔCt (n = 3)	Fold changes
Pothos green	ND	–	9.1 ± 1.9	1
Pothos albino	ND	–	21.9 ± 2.7	−7287.7
PG	9.3 ± 0.8	1	21.4 ± 0.8	−5093.8
V	11.1 ± 0.2	−3.7	22.3 ± 0.9	−9567.4
PY	11.8 ± 0.3	−6.2	22.9 ± 1.3	−13840.3

*The data represent the average of ΔCt compared to their internal control ± SD (n = 3). Fold change is determined as 2^(ΔΔct)^ and calculated using PG as 1 for CHL27, and pothos green control as 1 for EaZIP. Negative fold change indicates reduced gene expression. ND: not detected.

When ultra-structures were examined under the TEM, leaf cells from PG transgenic plants exhibited normal chloroplasts (Figure 7) but had fewer grana and more unstacked thylakoids compared to those of pothos green plants (Figure 6A). Leaf cells of V transgenic plants had completely developed chloroplasts, but only a few developed thylakoids. As for leaf cells of PY transgenic plants, they contained partially developed chloroplasts (Figure 7), which were different from those in wild-type albino leaf cells with only plastids (Figure 6B). Both partially developed chloroplasts in PY transgenic plants and plastids in wild-type albino plants did not have developed thylakoids. The numbers of thylakoids and the degrees of their stacking correlate with the chlorophyll *a* and *b* contents in these three types of *CHL27* transgenic plants, which agrees with the important roles of chlorophyll *a* and *b* in chloroplast development. These results prove that both defective color and dysfunctional chloroplast development in albino plants can be restored by compensating the loss of MPE cyclase.

## Discussion

The present study demonstrates that albino plants regenerated from *E. aureum* ‘Golden Pothos’ are long-lived when maintained in a modified MS culture medium under low light conditions (Figure 1). Albino plants occur in a wide range of plants including Arabidopsis (Ruppel et al., 2011), maize (Han et al., 1992; Hunter et al., 2018), barley (Hess et al., 1994; Li et al., 2019), rice (Liu et al., 2016; Zhang et al., 2016), tomato (García-Alcázar et al., 2017), Agave (Duarte-Aké et al., 2016; Us-Camas et al., 2017), *Artemisia vulgaris* (Knudson and Lindstrom, 1919; Spoehr, 1942), *Sequioa sempervirens* (Pittermann et al., 2018), *Cucumis sativus* (Yan et al., 2020), and *Pyrola japonica* (Shutoh et al., 2020). However, none was reported to have extended life span as the albino pothos. Although *S. sempervirens* and *P. japonica* can survive for a prolonged time, albino shoots of the former were derived from sprouts while the latter had symbiotic relationships with fungi. The longest individual albino plants survived on culture medium are Agave plants (∼5 years) (Duarte-Aké et al., 2016; Hernández-Castellano et al., 2020), followed by *A. vulgaris* with a life span of 3 months (Rischkow and Bulanowa, 1931) and maize plants of less than 4 months (Spoehr, 1942) while the former one should be long-lived. A consensus is drawn that individual albino plants are unable to perform photosynthesis; hence they die after stored energy is exhausted. Unlike albino pothos and Agave, most albino plants could not live long for years even though they are maintained on culture media with high sucrose (Dunford and Walden, 1991; Zubko and Day, 1998; Ruppel et al., 2011; Steiner et al., 2011; García-Alcázar et al., 2017), which raises a question concerning the involvement of other factors besides photosynthesis as a sole factor. One possible factor could be the life span of parental plants since most of them are annual and biennial species while ‘Golden Pothos’ plants are vegetatively growing and non-flowering perennial vines (Hung et al., 2016). Other factors could be the ability of plants to tolerate low light and absorb nutrients. Pothos plants are produced as houseplants for indoor decoration, and they are able to grow under low light conditions with limited nutrient supply (Chen et al., 2005). Other albino plants, such as *A. vulgaris* and maize inherently require high light intensity and abundant nutrient supply to grow. Once they are unable to conduct photosynthesis, they had little ability to absorb sugar and other nutrients from *in vitro* culture conditions as little increase in their fresh weight was observed (Rischkow and Bulanowa, 1931; Spoehr, 1942), thus they died in 3–4 months. Although albino pothos plants lost their ability to form roots during the continuous subculture, they grew well with a vigorous regeneration of new shoots in the modified MS culture medium, indicating that the albino pothos plants are able to absorb sugar and nutrients from the culture medium.

The albino pothos plants not only survived more than 11 years but also can be used for regeneration. Moreover, its active regeneration can be used for genetic transformation of a foreign gene with great efficiency. The defective color, dysfunctional chloroplast development and rooting capacity were restored by expressing Arabidopsis *CHL27.* The expression of *CHL27* resulted in the restoration of complete green as well as variegated plants. Pothos is among the most popular houseplants used in indoor conditions for decoration (Henny and Chen, 2003). Its interior use has been shown to abate indoor air pollutants (Sawada and Oyabu, 2008; Tada et al., 2010). Thus, there is increasing demand for new pothos cultivars around the world. However, due to its non-flowering nature, new cultivars cannot be developed through hybridization (Henny and Chen, 2003). Thus far, only four pothos cultivars in the market (Zhao et al., 2013a). The present study showed that using albino plants as materials, plants with different leaf colors can be produced, and the degree of color restoration is dependent on the level of *CHL27* expression. Additionally, these albino plants could also be used directly to express color related genes, such as Arabidopsis PAP1 (production of anthocyanin pigment 1) gene (*AtPAP1*) for red/purple color (He et al., 2017), and Chinese cabbage *BrMYB2* gene for purple color (He et al., 2020), to produce colorful plants. Thus, this established system could be used to generate different types of transgenic plants for developing new colorful pothos cultivars.

It is unclear at this time why the same transformation resulted in different types of plants. One possibility could be inappropriate coordination or interactions between the expressed foreign *CHL27* nuclear genes and other endogenous genes. This study show that overexpressing *EaZIP* failed to restore green color in transgenic plants (Figure 3). This failure could be attributed to the *EaZIP* cDNA without 5′-end sequence coding for the cTP. This *EaZIP* without cTP was initially cloned from green pothos through 5′RACE-PCR techniques (Hung et al., 2010). The expression of this *EaZIP* in green-leaved tobacco produced variegated transgenic plants (Guan et al., 2017). In addition to this cTP-lacking *EaZIP*, pothos might have a functional form of *EaZIP* with sequences encoding the *N*-terminal cTP. The interactions of *CHL27* with functional and non-functional *EaZIP* may cause the variation among the transgenic populations. Other possibilities could be epigenetic effects as reported in Agave plants (Duarte-Aké et al., 2016; Us-Camas et al., 2017). DNA methylation has been found to be intensive in pothos (Chen, unpublished data), it is possible that epigenetics through methylation is implicated the variation among regenerated plants. Future research is warranted to identify the underlying mechanisms.

As far as is known, this is the first report of successful stable transformation of an albino plant. In addition to be used for new pothos cultivar development, the albino plants along with the established transformation system could be valuable for studying critical events in chloroplast development. One example of such applications is to create inducible transgenic albino plants by controlling the expression levels of *CHL27* or a functional form of *EaZIP* under an inducible promoter. These inducible transgenic albino plants, which would keep white color under un-induced conditions but turn green under induced conditions, can be used as an experimental platform to study nuclear genes for plastid involved in chloroplast biogenesis and development. Those could be *E. aureum* genes or genes from different species with the exception of some species-specifically regulated. With this inducible platform, co-expression of a test gene with a fluorescent protein tag can be observed in co-transformed cells/tissues via live-cell imaging techniques (Wada and Suetsugu, 2004; Day and Davidson, 2009). Inducible co-transformed albino plants could be used not only to study the expression site(s) of the test gene but also to monitor the translocation process of its encoded protein during the chloroplast biogenesis under induced conditions. These albino plants could also be used to unravel biological questions, such as the effects of pigments on root development and light quality on plant development, where chlorophyll effects must be avoided or controlled as demonstrated previously (Jabben and Deitzer, 1979; Bavrina et al., 2002; Roelfsema et al., 2006; Van Norman et al., 2014). Recently, plants are becoming a promising expression system to produce a diverse range of biopharmaceuticals (Xu et al., 2012; Moon et al., 2020). Likewise, they can also be used to express high-value compounds and therapeutic proteins to facilitate downstream purification process without chlorophyll interference (Wilken and Nikolov, 2012; Mellor et al., 2018).

## Data Availability Statement

The original contributions presented in the study are included in the article/Supplementary Material, further inquiries can be directed to the corresponding author.

## Author Contributions

C-YH and JX conceived and designed the experiments. C-YH, JZ, CB, HL, FK, and XW performed the experiments. C-YH, CEO, KOB, JC, and JX analyzed the data. C-YH, JC, and JX wrote the article with contributions of all authors. All authors read and approved the manuscript, contributed to the article, and approved the submitted version.

## Conflict of Interest

The authors declare that the research was conducted in the absence of any commercial or financial relationships that could be construed as a potential conflict of interest.

## Supplementary Material

Supplementary Figure 1 Sequence alignment of the cDNAs of *EaZIP* (without cTP) (Accession #: FJ666046) and Arabidopsis *CHL27* (Accession #: NM_115553).
