# Supplementary figures and images for: Transformation of Long-Lived Albino Epipremnum aureum ‘Golden Pothos’ and Restoring Chloroplast Development
Source: Front Plant Sci. 2021 May 12;12:647507. doi: 10.3389/fpls.2021.647507 (PMC8149757; doi:10.3389/fpls.2021.647507)

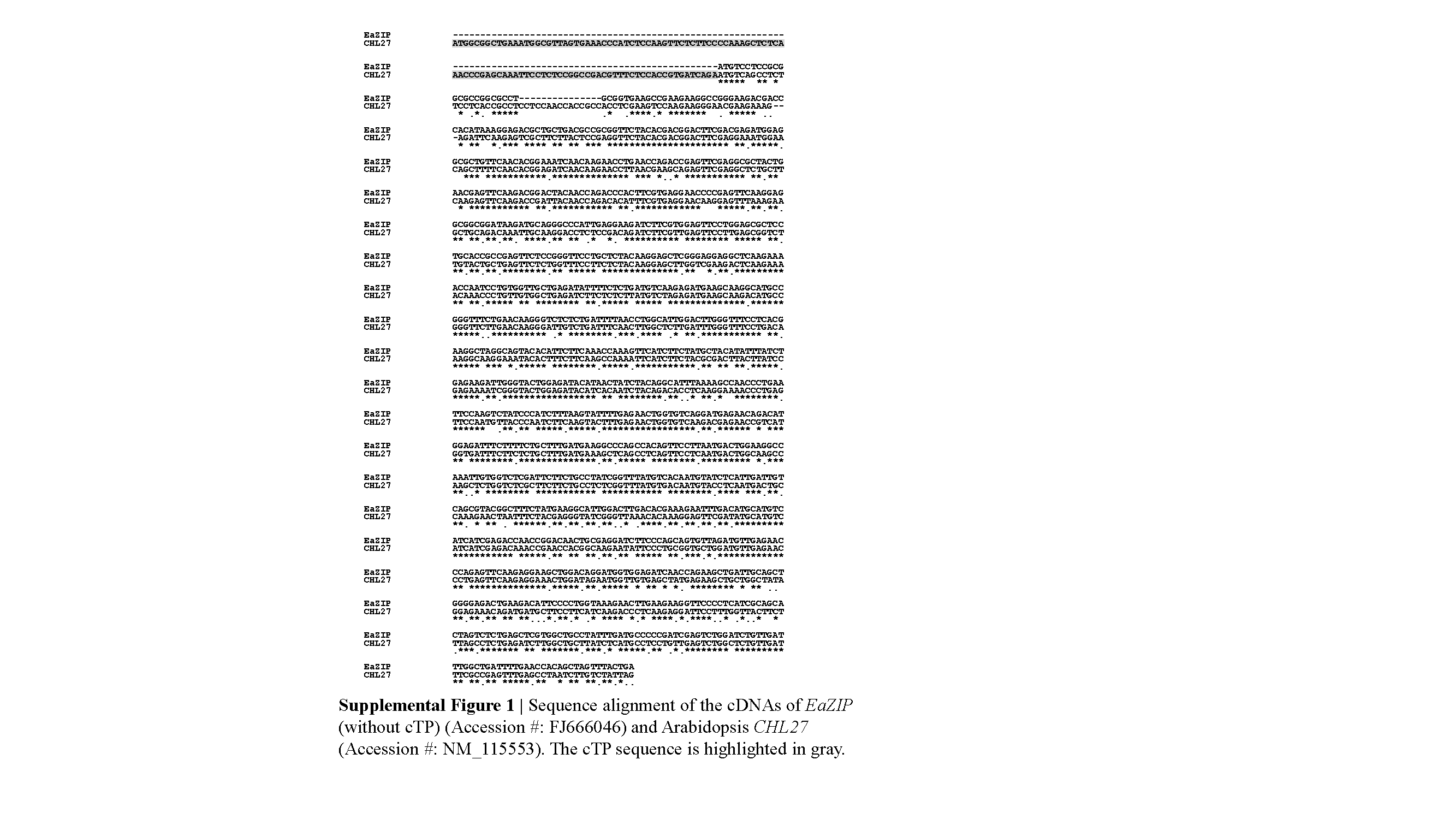

Supplement: Supplementary Figure 1 — Sequence alignment of the cDNAs of EaZIP (without cTP) (Accession #: FJ666046) and Arabidopsis CHL27 (Accession #: NM_115553). [file Image_1.TIFF]
